# Identification and Characterization of Novel Mutations in Chronic Kidney Disease (CKD) and Autosomal Dominant Polycystic Kidney Disease (ADPKD) in Saudi Subjects by Whole-Exome Sequencing

**DOI:** 10.3390/medicina58111657

**Published:** 2022-11-16

**Authors:** Othman R. Alzahrani, Hanan E. Alatwi, Amnah A. Alharbi, Abdulrahman H. Alessa, Osama M. Al-Amer, Abeer F. R. Alanazi, Anwar M. Shams, Esra’a Alomari, Abdallah Y. Naser, Faisal a. Alzahrani, Salman Hosawi, Saeed M. Alghamdi, Wed A. Abdali, Imadeldin Elfaki, Yousef M. Hawsawi

**Affiliations:** 1 Department of Biology, Faculty of Sciences, University of Tabuk, Tabuk 71491, Saudi Arabia; 2 Genome and Biotechnology Unit, Faculty of Sciences, University of Tabuk, Tabuk 71491, Saudi Arabia; 3 Department of Biochemistry, Faculty of Sciences, University of Tabuk, Tabuk 71491, Saudi Arabia; 4 Department of Medical Laboratory Technology, Faculty of Applied Medical Sciences, University of Tabuk, Tabuk 71491, Saudi Arabia; 5 Department of Pharmaceutical and Biological Sciences, UCL School of Pharmacy, London WC1E 6BT, UK; 6 Department of Pharmacology, College of Medicine, Taif University, P.O. Box 11099, Taif 21944, Saudi Arabia; 7 Department of Applied Pharmaceutical Sciences and Clinical Pharmacy, Faculty of Pharmacy, Isra University, Amman 11622, Jordan; 8 Department of Biochemistry, Faculty of science, Embryonic Stem Cell Unit, King Fahad Center for Medical Research, King Abdulaziz University, Jeddah 21589, Saudi Arabia; 9 Department of Biochemistry, Faculty of Science, King AbdulAziz University, Jeddah 21589, Saudi Arabia; 10 Artificial Intelligence in Systems Biology Research Unit, The King Abdulaziz University and The University of Oxford Center for Artificial Intelligence for Precision Medicine, Jeddah 21589, Saudi Arabia; 11 Department of Medicine, King Faisal Specialist Hospital and Research Centre, P.O. Box 40047, Jeddah 21499, Saudi Arabia; 12 Research Center, King Faisal Specialist Hospital and Research Center, MBC J04, P.O. Box 40047, Jeddah 21499, Saudi Arabia; 13 College of Medicine, Al-Faisal University, P.O. Box 50927, Riyadh 11533, Saudi Arabia

**Keywords:** whole-exome sequencing, autosomal recessive polycystic kidney disease (ARPKD), autosomal dominant polycystic kidney disease (ADPKD), polycystin-1-polycystin-2 *PKD1*, *PKD2*, end-stage renal disease ESRD

## Abstract

*Background:* Autosomal dominant polycystic kidney disease (ADPKD) is a condition usually caused by a single gene mutation and manifested by both renal and extrarenal features, eventually leading to end-stage renal disease (ESRD) by the median age of 60 years worldwide. Approximately 89% of ADPKD patients had either *PKD1* or *PKD2* gene mutations. The majority (85%) of the mutations are in the *PKD1* gene, especially in the context of family history. Objectives: This study investigated the genetic basis and the undiscovered genes that are involved in ADPKD development among the Saudi population. *Materials and Methods:* In this study, 11 patients with chronic kidney disease were enrolled. The diagnosis of ADPKD was based on history and diagnostic images: CT images include enlargement of renal outlines, renal echogenicity, and presence of multiple renal cysts with dilated collecting ducts, loss of corticomedullary differentiation, and changes in GFR and serum creatinine levels. Next-generation whole-exome sequencing was conducted using the Ion Torrent PGM platform. *Results:* Of the 11 Saudi patients diagnosed with chronic kidney disease (CKD) and ADPKD, the most common heterozygote nonsynonymous variant in the *PKD1* gene was exon15: (c.4264G > A). Two missense mutations were identified with a *PKD1* (c.1758A > C and c.9774T > G), and one patient had a *PKD2* mutation (c.1445T > G). Three detected variants were novel, identified at *PKD1* (c.1758A > C), *PKD2L2* (c.1364A > T), and *TSC2* (deletion of a’a at the 3’UTR, R1680C) genes. Other variants in *PKD1L1* (c.3813_381 4delinsTG) and *PKD1L2* (c.404C > T) were also detected. The median age of end-stage renal disease for ADPK patients in Saudi Arabia was 30 years. *Conclusion:* This study reported a common variant in the *PKD1* gene in Saudi patients with typical ADPKD. We also reported (to our knowledge) for the first time two novel missense variants in *PKD1 and PKD2L2* genes and one indel mutation at the 3’UTR of the *TSC2* gene. This study establishes that the reported mutations in the affected genes resulted in ADPKD development in the Saudi population by a median age of 30. Nevertheless, future protein–protein interaction studies to investigate the influence of these mutations on *PKD1* and *PKD2* functions are required. Furthermore, large-scale population-based studies to verify these findings are recommended.

## 1. Introduction

Monogenic cystic kidney disease is a cilia-associated disorder that is composed of two forms: autosomal recessive polycystic kidney disease (ARPKD) and autosomal dominant polycystic kidney disease (ADPKD) [1]. ARPKD is a rare and severe form of the disease that mainly presents in children, while ADPKD is the most common hereditary genetic renal disorder that occurs primarily in adulthood [2]. Clinically, the two forms of the disease can be distinguished based on the presence of many criteria, such as kidney morphology, location of the cyst, hepatic fibrosis, arterial hypertension, and age at the presentation of the symptoms [3]. The majority of ADPKD patients presented with hypertension and deterioration in the glomerular filtration rate (GFR) [4], which might require renal dialysis or transplantation in the advanced stages [5]. Based on several clinical studies, ADPKD is the most prevalent genetic renal disorder, with estimated cases of 1 in 500-2500 individuals [6] anticipated to affect over 10 million individuals globally from various ethnicities; thus, it is one of the significant clinical concerns [1].

Numerous biological and clinical studies have been conducted to delineate the genetic mechanism of ADPKD [2,7,8,9]. Mutation in the genes *PKD1* (78% of cases) or *PKD2* (15% of cases) accounts for the most common genetic alterations involved in the pathogenesis of ADPKD [10]. The *PKD1* gene is located on chromosome 16 and encodes polycystin-1 protein (PC1), a large transmembrane integral glycoprotein [10]. The *PKD1* gene consists of 46 exons with a large, duplicated region from exons 1-33 that shares a high degree of sequence identity with the other six pseudogenes near *PKD1* on the same chromosome (16p) [11]. The *PKD2* gene is located on chromosome 4 and encodes polycystin-2 protein (PC2), a transmembrane protein. *PKD2* is smaller than *PKD1* and has only 15 exons [11]. Although mutations of *PKD1* and *PKD2* genes are fully penetrant, their expressivity is associated with different cases of variable severity in members of the same family, suggesting the presence of additional gene modifiers [12].

Clinical information combined with imaging-based strategies, preferentially ultrasonography, is the primary approach to diagnosing patients with ADPKD [13,14]. MRI or CT scans with contrast provide highly specific and sensitive modalities in calculating the number and diameter of the cysts, especially in high-risk younger people with positive family history [15]. Aside from imaging techniques, genetic testing is required to confirm the definitive diagnosis of ADPKD, particularly in young members with few cysts and no family history or when the diagnosis is vague [8,16,17]. Indeed, genetic testing affords a valuable diagnostic and prognostic tool that might further improve clinical management and outcomes in cystic kidney disease patients [2,17,18]. Over 1500 mutations with high allelic heterogeneity of *PKD1* and *PKD2* genes have been indicated in the ADPKD dataset [1]. Nevertheless, the detection of *PKD1* mutation requires comprehensive analysis because of its large size, complex genomic structure, and expensive technique [19].

Transmission of genetic susceptibility diseases, such as inherited renal disorders, was found to be more frequent in areas where consanguineous marriages are common, such as in the Kingdom of Saudi Arabia (KSA) [20]. Since the rate of consanguinity marriages in KSA was reported to reach up to 55%, homozygous mutations in the *PKD1* gene and other genes related to cystic kidneys would be more anticipated [21]. Furthermore, allelic and genetic heterogeneity for many Mendelian disorders has evolved in KSA [22]. An observational study conducted in Saudi Arabia reported a high incidence of genetically inherited kidney diseases such as polycystic kidney disease (PKD), familial juvenile nephronophthisis, congenital urological anomalies, and familial nephrotic syndrome among Saudi children [23]. Moreover, antenatal analysis using an ultrasonography test along with Sanger sequencing of the *PKD1* gene was employed among four consanguineous Saudi families to investigate the genetic background of the early-onset PKD [24]. Additionally, homozygous and compound heterozygous hypomorphic *PKD1* missense alleles mutation was detected in two families and resulted in the early onset of an aggressive disease yet was compatible with life [24]. Al-Muhanna et al. performed the exome sequencing using WES technology on 16 Saudi ADPKD patients and reported that variants related to *PKD1* and *PKD2* genes are the most frequently detected mutations in Saudi patients with typical ADPKD. On the other hand, a specific form of ADPKD was associated with rare pathogenic mutations in cytogenic genes such as *PKHD1*, *PKD1L3*, *EGF*, *CFTR*, and *TSC2* [25].

ADPKD is considered a critical health issue in Saudi Arabia, and progression to end-stage kidney disease (ESKD) is inevitable. Numerous reports have exemplified the genetic elements involved in the development of PKD/ADPKD among the Saudi population [20,21,23,24,25,26,27]. Yet the extent to which these genetic variants are involved in the pathogenesis, erraticism in clinical scenarios, and disease severity remain poorly understood. Previously, we reported several mutations that are associated with diseases in Saudi Arabia [27,28,29,30,31]. Therefore, the current study aims to examine the genetic basis and the hitherto undiscovered genes that might be involved in developing ADPKD among the Saudi population.

## 2. Methods, Materials, and Subjects

### 2.1. Patients and Family Recruitment

This study included 11 patients from different families, diagnosed with ADPKD and chronic kidney disease (CKD) probands ascertained in the nephrology clinic at the King Faisal Specialist Hospital and Research Center Jeddah (KFSH & RC); participants were enrolled in the western province of Saudi Arabia. All subjects signed the informed consent and documentation of family history forms. The diagnosis of ADPKD was achieved based on different criteria, and measurements comprised (1) medical history: the patient’s clinical manifestations; in addition to the presence of positive family history, (2) diagnostic images: CT images include enlargement of renal outlines, renal echogenicity, presence of multiple renal cysts with dilated collecting ducts, and loss of corticomedullary differentiation; and (3) laboratory data: evaluation of glomerular function (GFR) and creatinine level in the serum. Demographic data retrieved from the patients’ medical reports, including age, gender, race, ethnicity, and previous and current medical history (hypertension status, CKD stage, eGFR, affected family member, and presence of other medical conditions), are presented in Table 1. Patients were classified as G1–G5 based on the eGFR and A1–A3 based on the albumin:creatinine ratio (ACR). 

### 2.2. Ethical Approval and Participants’ Consent

This study was approved by the Institutional Review Board of King Faisal Specialist Hospital and Research Center Jeddah (IRB number 2018-36) on 18 September 2018. Informed consent for the retention and use of patient data for research purposes was routinely obtained at the time of obtaining consent for the procedures. Before blood sample collection, all patients who met the inclusion and exclusion criteria completed the informed consent (ICF) and case report form (CRF).

### 2.3. DNA Extraction

Peripheral blood samples were collected from each patient (approximately 2–3 cc). Subsequently, the genomic DNA was extracted from all blood samples using a Gentra Puregene Blood Kit (Qiagen, Cat No: 158389, Düsseldorf, Germany) according to the manufacturer’s instructions. Briefly, 20 µL of proteinase K was added to 200 µL of whole blood, mixed, and incubated for 1 min at room temperature (15–25 °C), followed by adding 200 µL of buffer to the sample. Then, the mixture was incubated in a water bath at 56 °C for 10 min and spun for 2 min at 2000× *g*, and 200 μL of ethanol (96–100%) was added to the sample, followed by mixing by pulse-vertexing for 15 s. Finally, 100 µL of the DNA Hydration Solution was added, and it was vortexed for 5 s and incubated at 65 °C for 5 min. The quantity and quality of extracted DNA were evaluated by using the Qubit 2.0 Fluorometer (Life Technologies, Waltham, MA, USA) with the Qubit dsDNA High Sensitivity Assay Kit (Life Technologies, Cat No Q32851, Waltham, MA, USA) and NanoDrop 2000 system (Thermo Scientific, Waltham, MA, USA), respectively, according to the manufacturer’s instructions.

### 2.4. DNA Library Preparation

Extracted DNA (100 ng per sample) was used for library preparation using the Ion AmpliSeq Library Kit Plus (Life Technologies, Cat # 4488990, Waltham, MA, USA) with the Ion AmpliSeq Exome RDY Kit (Life Technology, cat # 8849838, Waltham, MA, USA) according to the manufacturer’s instructions.

### 2.5. Next-Generation Sequencing (NGS) Technology

DNA was sequenced by utilizing massive parallel sequencing via Ion Torrent PGM with the Ion PI Hi-Q Sequencing 200 Kit (Life Technologies, Cat# 4488651, Waltham, MA, USA) and the Ion PI Chip Kit v3 (Life Technologies, Cat# A26770, Waltham, MA, USA) according to the manufacturer’s instructions. Briefly, the prepared DNA was loaded in Ion Torrent semiconductor chips and loaded into the Ion Torrent PGM machine.

### 2.6. Variant Filtration

The reads of the Ion Torrent sequencing machine (Life Technologies, Waltham, MA, USA) were aligned to the hg19 reference genome through the tMap program. The aligned reads were investigated for variant calling through the Torrent Suite Variant Caller TVC program. The variants were annotated by using public and in-house databases and the Saudi Human Genome program

### 2.7. In Silico Analysis

Multiple in silico algorithm software programs were used to determine the structural function and to evaluate the pathogenic effect and the impact of the specific mutation on the biological processes of the protein, such as Mutation taster [32], Polyphen-2.0 [33], SIFT [34], and PROVEAN [35].

Polymorphism Phenotyping v2 (PolyPhen-) was used as a tool to predict the possible impact of an amino acid substitution on the structure and function of the reported protein using straightforward physical and comparative considerations (http://genetics.bwh.harvard.edu/pph2/ (accessed on 18 January 2022)

### 2.8. Bioinformatics

The mapping of the mutations to protein domains was performed manually using the NCBI Nucleotide database and UNIPROT (https://www.uniprot.org (accessed on 18 January 2022)). The protein–protein interaction analysis was performed using the STRING database (https://string-db.org (accessed on 18 January 2022)) and Cytoscape (https://cytoscape.org (accessed on 18 January 2022)) for data retrieval and merging, respectively. The gene ontology [36,37] and KEGG (https://www.genome.jp/kegg/pathway.html (accessed on 18 January 2022)) pathway analyses were performed using STRING enrichment. All networks retrieved from STRING in this work had a confidence score of 0.4 and included all types of evidence available in the database.

### 2.9. Statistical Analysis

Statistical analysis was conducted via SPSS version 22.0 statistical package software (SPSS, Chicago, IL, USA). All probability values (*p*-values) below 0.05 were considered statistically significant. The ORs and 95% CIs were calculated using the chi-squared test to study the comparison of genetic variations.

## 3. Results

### 3.1. Basic Clinical Characteristics and Genetic Analysis of the Studied Subjects

In this study, 11 patients clinically diagnosed with ADPKD and CKD were selected from the King Faisal Specialist Hospital and Research Center from 2002 to 2019 to participate in this work.

**Figure 1:**
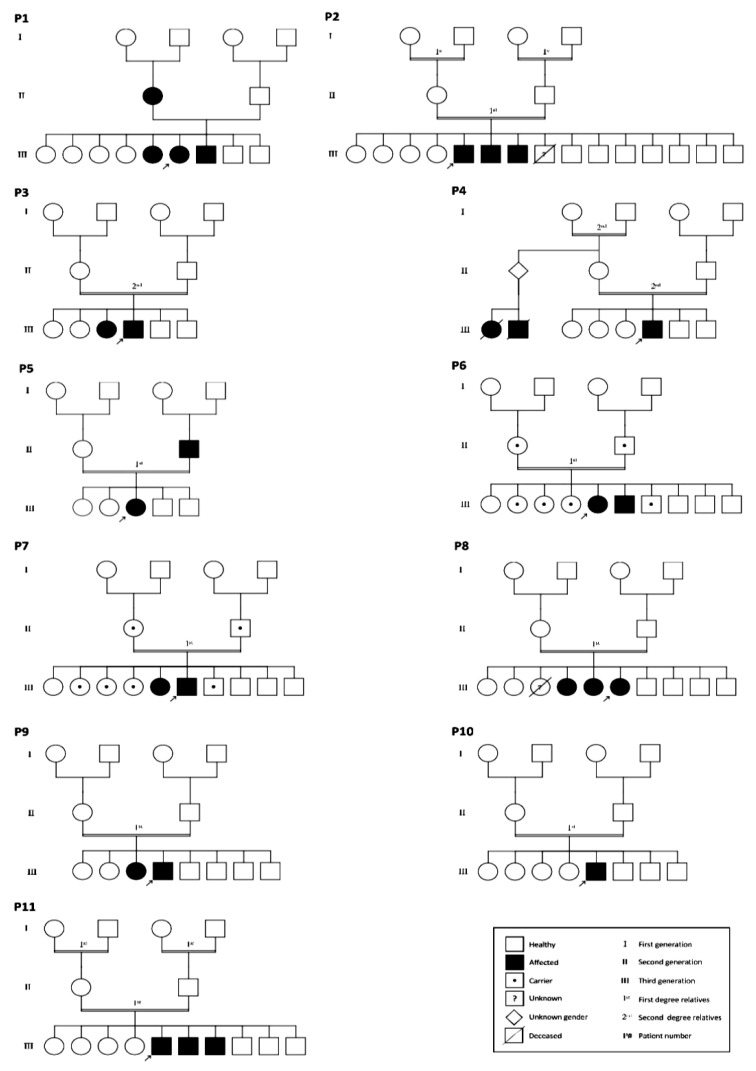
Familial pedigree of the subjects. Three-generation pedigrees for 11 patients diagnosed with ADPKD (P1–11). Affected individuals are indicated by black; index cases are characterized by a black arrow, square (male), and circle (female). Pedigree Chart Designer was used for this figure.

As shown in Table 1, patients’ ages were distributed among young and middle-aged categories (18–46 years old), which included four females and seven males. All patients presented with different stages of CKD and abnormally low eGFR values and were diagnosed with the presence of other kidney disorders. Hypertension was detected in 63.6% (7/11) of the participants. Positive family history was detected in all participants except one patient (90.90%). We excluded from the study all patients with syndromic causes of multiple renal cysts, such as von Hippel–Lindau disease, tuberous sclerosis, and familial polythelia with multiple renal cysts. Furthermore, the family history including affected and carrier family members with ADPKD is presented in the familial pedigree for each patient (Figure 1).

### 3.2. Mutations Identified in Patients with ADPKD

To characterize the genetic alteration that might be engaged in the pathogenesis of ADPKD in the selected individuals, we used NGS technology on the extracted DNAs. Among eleven cases, five missense mutations (45.45%) were identified in the *PKD1* gene. Three out of these five mutations were identified in exon15: c.4264G > A: p.A1422T, including the following patients P2, P8, and P11. In the following patients, the other two mutations were detected in exon9: c.1758A > C: p.E586D and exon29: c.9774T > G: p.F3258L, P4 and P9, respectively. One missense mutation (9.09%) in the *PKD1L2* gene was identified in exon2: c.404C > T: p.P135L in P5, and another missense mutation (9.09%) in the *PKD2L2* gene was identified in exon9: c.1364A > T: p.N455I in P6. Additionally, one missense mutation (9.09%) in the *PKD2* gene was identified in exon6: c.1445T > G: p.F482C in the third patient (P3). The first patient showed one non-frame-shift substitution in the *PKD1L1* gene in exon 24: c.3813_3814delinsTG. In the seventh patient, we detected exonic alteration in exon 39: c.5038C > T: p.R1680C in the *TSC2* gene. The last patient (P10) shows no genetic abnormalities or positive family history (Table 1 and Table 2). To further determine the contribution of the identified mutations to the development and predisposition to ADPKD, we used the predictable bioinformatics tools, including Mutation taster, Polyphen-2.0, SIFT, and PROVEAN, to estimate the pathogenicity score for each detected genetic alteration. The pathogenic effects were observed when the mutation variants happened in the *PKD1* gene involving exons 9 and 29, *PKD2* gene in exon3, *PKD2L2* in exon9, and *TSC2* gene in exon39 (showed likely pathogenicity). To provide insight into the impact of these identified mutations on the proteins’ stability and functions, we manually mapped each genetic mutation to its corresponding protein using the NCBI Nucleotide database and UNIPROT software. Our analyses revealed that two *PKD1* gene mutations did not localize within any of its known protein domains or post-translational modification (PTM) sites; these mutations were detected in exon9: c.1758A > C: p.E586D and exon29: c.9774T > G: p.F3258L. On the other hand, we found that the third mutation of the PDK1 gene (exon15: c.4264G > A: p.A1422T) is located within the PKD9 domain of PC-1 protein. Furthermore, the missense mutations of *PKD1L2* and *PKD2L2* genes located on exon2: c.404C > T: p.P135L and exon9: c.1364A > T: p.N455I were found to be positioned within two different motifs of PC-1 protein: the C-type lectin domain and the receptor for egg jelly (REJ), respectively. The missense mutation of the *PKD2* gene (exon6: c.1445T > G: p.F482C) was not placed on any known protein domains of PTM sites of that protein. Finally, the mutation (exon39: c.5038C > T: p.R1680C) in the *TSC2* gene was localized within its Rap-GAP domain.

### 3.3. Bioinformatics Analysis and Data Mining Highlight Potential Proteins Interactions between PKD1 and TSC2 via BRSK2

**Figure 2:**
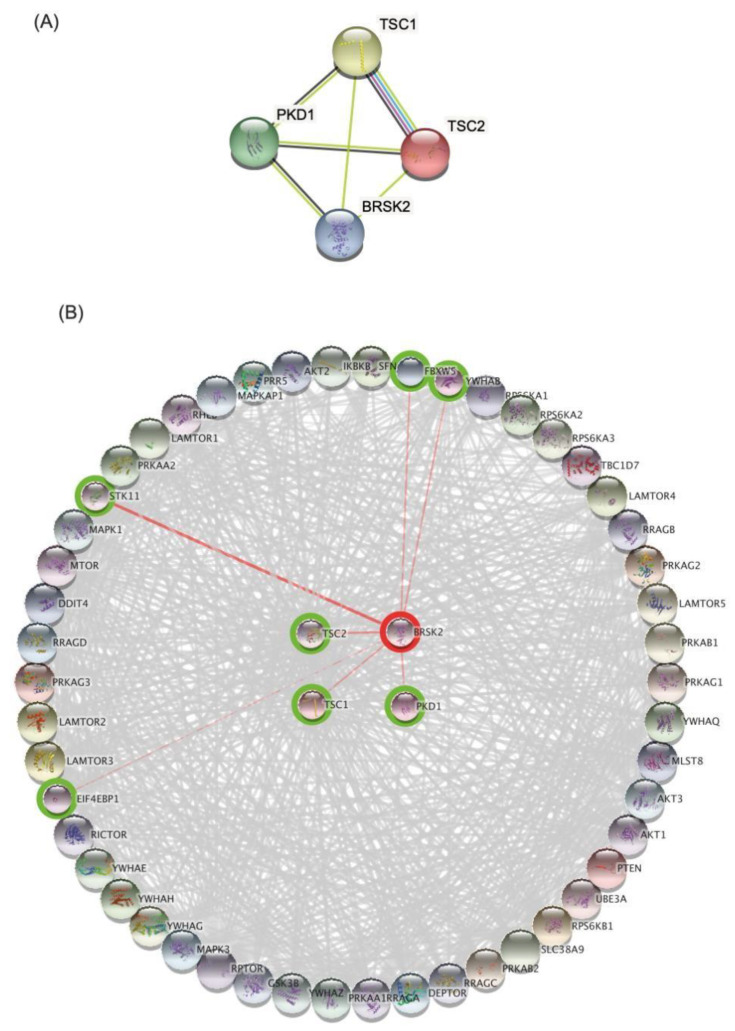
Prediction of protein–protein interaction (PPI) network analysis of proteins harboring the identified mutations (**A**) the PPI network of the mutated protein. (**B**) The expansion of the network to contain up to 50 more partners based on the various interactions offered by STRING with a confidence score of 0.4.

**Figure 3:**
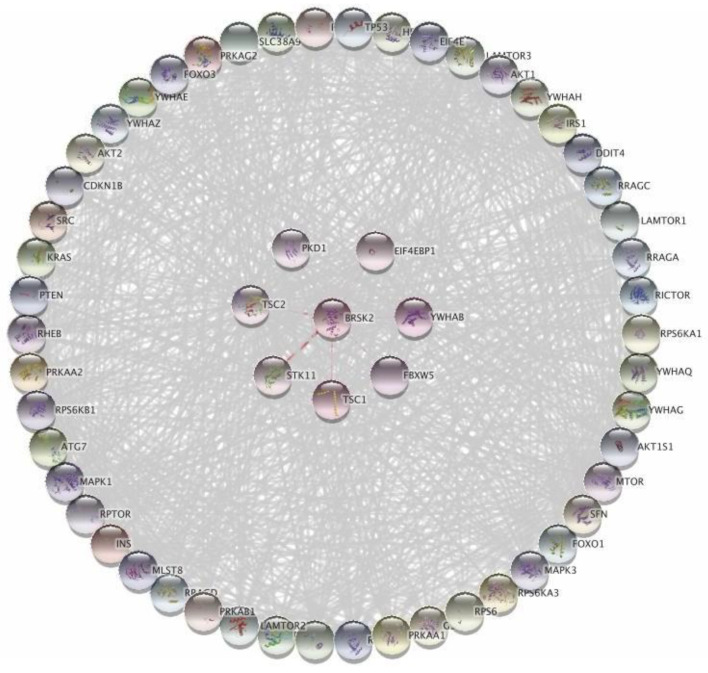
Protein-protein interaction network analysis of the subnetwork focused on *BRSK2*. The first neighbors of *BRSK2* from the main network (Figure 2B) included 7 proteins that were used to form different levels of interactions, which was later expanded to include 50 more interactions in the second level. These interactions are based on the various interactions offered by STRING and a confidence score of 0.4.

To investigate potential interactions between the different proteins possessing the identified mutations that might eventually contribute to the pathogenesis of ADPKD, the protein–protein interaction (PPI) network for each of the targets (*PKD1*, *PKD2*, *TSC2*, and *PKD1L2*) was retrieved from the STRING database. As shown in Figure 2A, a map composed of four different proteins (*PKD1*, *TSC1*, *TSC2*, and *BRSK2*) resulted from merging various PPI networks. Each protein represents a common intersecting point of these merging networks. To gain further insight into the different biological activities of these four proteins, we expanded the PPI network of the four proteins to include 50 more interactions with medium interaction confidence scores of 0.4. Next, we applied gene ontology (GO) and KEGG pathway enrichment analyses for the entire network (Figure 2B). Most top nodes were influenced by *TSC1*, *TSC2*, and *PKD1*, while *BRSK2* was found among significant nodes but at a lower ranking. To further elucidate the contribution of *BRSK2*, a subnetwork from Figure 2B was created by selecting the first neighbors of *BRSK2*, including *TSC2*, *PKD1*, *TSC1*, *FBXW5*, *EIF4EBP1*, *STK11*, and *YWHAB* (Figure 3) for assessment by GO and *KEGG* pathway analyses. The generated map revealed similar interacting nodes to that one obtained in the primary network (Figure 2B). Yet, the new network showed different arrangements of the nodes based on significance, such as the *PI3K* and *AMPK* signaling pathways. Next, we aimed to explore the potential role of *BRSK2* in *ADPKD* pathogenesis. Therefore, a search process on PubMed was conducted, where *BRSK2* has searched along with different terminologies such as *ADPKD*, *ARPKD*, *PKD*, Cyst, and kidney. Interestingly, searching *BRSK2* against mTOR yielded several hits describing the involvement of the BRSK2 protein and *TSC2* in the regulation of mTOR signaling in different contexts, including cancer cell survival and development [38]. Moreover, the PI3K/AKT/mTOR pathway was reported to encourage the growth of renal cysts and increase their size and proliferation [39].

### 3.4. Mutation and Allele: Frequency

For the missense SNP (rs140980374), G/A/Ancestral: G|MAF: < 0.01 (A), highest population MAF: < 0.01. For the missense variant in SNP (rs201455881), The 1000 Genomes Project Phase 3 allele frequencies showed allele frequencies of G: 0.9998 and A: 0.0001. The genotype frequency is G/G: 0.9996 and A/G: 0.00039 in all populations.

## 4. Discussion

### 4.1. Identification of Novel Variants with a Potential Contribution to the Evolution and Pathogenesis of ADPKD in Saudi Patients

ADPKD accounts for the most common genetic kidney diseases worldwide and is considered a significant health concern and clinical challenge [2,6]. Among the Saudi population, ADPKD is one of the main hereditary medical conditions due to the high level of consanguineous marriages, a suitable setting that encourages the transmission of inherited disorders [21,22]. Furthermore, the incidence of novel mutations that might contribute to the early onset of an aggressive form of ADPKD was also reported to be frequent among the Saudi population [24,25,26,40]. We previously identified several variants that are associated with diseases in the Saudi population [41,42,43,44,45]

Therefore, in the current work, we aimed to expand our knowledge and further understand the genetic background engaged in ADPKD evolution, pathogenesis, and progression, thus facilitating the insinuation of appropriate, timely management. To achieve this goal, we used NGS technology to screen the whole-exome variants of 11 clinically diagnosed ADPKD patients belonging to different Saudi families. Our analysis revealed that most of the obtained mutations in the selected patients were detected in *PKD1* or *PKD2* genes (54.54%), in agreement with the previous Saudi reports indicating that most individuals with ADPKD (89.1%) had mutations in the *PKD1* or *PKD2* genes [25,26,41]. Moreover, our results also showed that patients who harbor either *PKD1* or *PKD2* gene mutations showed an earlier age of diagnosis (18–37 years) with a median of 28 years, suggesting the substantial involvement of positive family history in the occurrence of the disease [46,47]. Additionally, 50% of the studied PKD1/PKD2 affected individuals reached a high disease grade (G4/5) or end-stage renal disease (ESRD).

Several reports have shown that the detection of various types of mutations in *PKD1* and *PKD2* genes was associated with rapidly progressive disease and early onset of renal dialysis, eventually yielding ESRD [48,49,50,51,52,53]. Moreover, individuals with *PKD1* mutation developed ESRD at a median age of 58, while *PKD2* mutation patients showed better prognosis and delayed onset of ESRD at around 80 years [54,55]. Interestingly, our finding documented three novel mutations in the following genes *PKD1*, *PKD2L2*, and *TSC2* of the Saudi ADPKD patients.

The first novel missense variant was detected in patient P4 at position p.E586D of the PKD1 protein, and it was predicted by SIFT and PolyPhen programs, with pathogenic effects on the structure and function of the protein. The second novel missense variant was located at position p.N455I of the PKD2L2 protein in patient P6. Its pathogenic effect was estimated to be of uncertain significance using SIFT and PolyPhen programs. It was found that protein variants occurring at promoter regions or the 3’UTR could affect gene splicing, transcription factor binding, or interfere with microRNA binding sites, which could produce an abnormal amount of protein [56]. Here, we also documented a third novel variant residing at the 3’UTR of the *TSC2* gene in patient P7 at the position of 2138671–2138672 on chromosome 16, which corresponds to the protein site p.R1680C, which resulted in amino acid deletion and showed likely pathogenetic effects. *TSC2*, the tuberous sclerosis complex, the most common gene for an autosomal dominant genetic disease, is featured by the formation of benign tumors (hamartomas) in different organs [57]. Tuberous sclerosis manifests in the presence of CNS/mental, psychological, and skin manifestations and frequent renal cysts [58,59]. *TSC2* and *PKD1* genes were demonstrated to lie adjacent at chromosome 16p13.3, highlighting a shared cross-talk by both genes [58]. High-throughput genetic tools allowed the discovery of a broad range of mutations in *TSC1* or *TSC2 genes* [59]. A study found that large mutational deletion of the *TSC2* gene is correlated with the development of early-onset polycystic kidney disease in which the adjacent *PKD1* gene was also affected by this deletion [60]. Likewise, Consugar et al. reported that large genomic deletions could also cause *PDK1*/*TSC2* contiguous gene deletion syndrome that is characterized by the presence of various degrees of cystic kidney with early onset of ESRD, hypertension, neurological, behavioral, facial, and skin abnormalities [57,61,62]. Additionally, individuals with a severe and early diagnosed form of the renal cystic disease were also found to display *TSC2* gene mutation [58].

### 4.2. Potential Damaging Impacts of the Detected Mutations on the Protein Structure and Function

To further broaden our understanding of the detrimental influences of the identified mutations on the protein functional efficiency, we calculated the pathogenicity score and then elucidated the positions at which these different mutations are situated within each protein. Five patients with *PKD1* gene mutations were identified; three of them (P2, P8, and P11) display a missense variant (exon15: c.4264G > A: p.A1422T) in which the alanine (a very small hydrophobic) is replaced by threonine (a small neural amino acid). This missense variant (exon15: c.4264G > A: p.A1422T) showed an uncertain pathogenic effect and was estimated to locate within the PKD9 domain of the PC-1 protein. *PKD1* gene encodes polycystin-1 (PC-1), a large cell surface glycoprotein. A 16-PKD-repeat region (or Ig-like domain) was identified within the extracellular region of the PC-1 protein [63]. The PKD domain showed a ligand binding motif for potential regulation of extracellular signaling involved in protein–protein and protein–carbohydrate interactions and cellular adhesive process [64,65]. Indeed, antibody targeting of the PKD domain in an in vitro model interfered with cellular interactions, thus suggesting that maintaining an intact PKD domain is an important step in preventing cystic generation in ADPKD [66].

The other two patients, P4 and P9, showed missense mutations in exon9: c.1758A > C: p.E586D and exon29: c.9774T > G: p.F3258L, respectively. In the first mutation c.1758A > C: p.E586D, the glutamic acid (medium, hydrophilic, and negatively charged) is replaced by aspartic acid (small, hydrophilic, negatively charged). In the second mutation, c.9774T > G: p.F3258L, the phenylalanine (very large, hydrophobic) is replaced by leucine (large, hydrophobic). These two variants were calculated to possess a pathogenic impact on protein function, yet they were not situated at any predetermined domain or PTM site of the protein. Along with this, the missense mutation of the *PKD2* gene (exon6: c.1445T > G: p.F482C) revealed the same findings, and this mutation was also not found to be located on any known protein domains of PTM sites. The physiological roles of ADPKD proteins, PC-1, and PC-2, are regulated by several PTM processes, including phosphorylation, glycosylation, and proteolytic cleavage [67].

Our results also revealed the uncertain pathogenic significance of both mutations of *PKD1L2* and *PKD2L2* (exon2: c.404C > T: p.P135L and exon9: c.1364A > T: p.N455I). In the mutation of exon2 c.404C > T: p.P135L, the proline (small neutral) is replaced by leucine (large, hydrophobic), and in exon9: c.1364A > T: p.N455I, the asparagine (small, hydrophilic) is replaced by isoleucine (large and hydrophobic). These variants were found to be positioned within two distinct protein domains, the C-type lectin and REJ domains of PC-1 protein on its ECD, respectively. Additionally, C-type lectins were found to modulate cell–cell adhesions and various immunological responses to conduct intracellular signaling [68,69]. The C-type lectin domain, through an auto-proteolytic process, can be fragmented from the PC-1 protein and cell membrane to act as secreted ligand to activate the polycystine channels in the cilia and plasma membrane [70]. Thus, the mutation of *PKD1L2* (exon2: c.404C > T: p.P135L) at the C-type lectin domain could affect this motif’s function and consequently contribute to the disease development. Numerous mutations, including missense mutations, have been identified in this motif, with the majority of these variants predicted to deliver detrimental consequences [71]. Variants, including the REJ region, disrupt the cleavage ability and block the activation of the JAK-STAT pathway by PC-1 protein and inhibit tubular formation, a cardinal feature of PC-1. Indeed, an in vitro model of transfected cells with the mutated REJ domain produced spherical cyst-like structures, thus highlighting the substantial involvement of this motif in ADPKD pathogenesis [72]. Together, these results indicate the indispensable role of PC-1 protein in preserving functional kidneys and that its N-terminal domain (ECD) is a hotspot for various mutations that engaged in ADPKD development.

The TSC1 C-domain interacts with the TSC2 N-domain to generate the TSC complex with a gene product of hamartin and tuberin, respectively [73]. Interestingly, PC-1 protein was also found through its cytoplasmic C-terminal to inhibit mTOR activity by direct interaction with TCS2 and altering its cellular localization, thus preventing TSC2 inactivation by AKT phosphorylation. Additionally, PC-1/C-domain enhances the interaction of TSC2 with its partner TSC1 which further suppresses the mTOR cascade [74]. Immunohistochemistry studies showed low expression of tuberin (TSC2) in angiomyolipoma, which contains activated mTOR signals, compared to healthy kidneys, authenticating the suppression role of TSC2 on mTOR activity [75]. Additionally, compelling evidence has shown that the overactivation of mTOR signaling encourages cyst growth and proliferation in PKD [40] and that epithelial cells of cystic kidneys in ADPKD express elevated levels of mTOR activity [76]. Rapamycin, an mTOR inhibitor, showed promising results in reducing kidney volume by up to 25% in ADPKD patients [77]. Likewise, dual blocking of mTOR using NVP-BEZ235 treatment resulted in normalizing renal function and morphology in ADPKD animal models [78]. The mutation described in the current study of the *TSC2* gene (exon39: c.5038C > T: p.R1680C) was estimated to be placed within TSC2/RAP-GAP domain and was predicted to show a potential pathogenic effect. This variant potentially plays a significant role alongside other mutations in the pathogenesis and progression of ADPKD disease.

### 4.3. Prediction of Protein–Protein Interactions Demonstrated a Common Pathogenic Pathway

**Figure 4:**
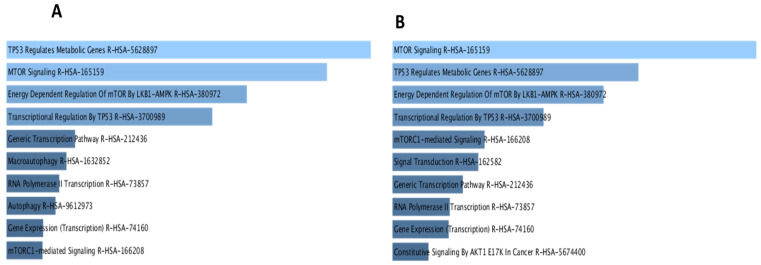
Gene ontology and KEGG pathway analysis of the different PPI networks. (**A**) Top 10 KEGG pathways of the expanded network based on the identified PPI network for the mutated proteins (PKD1, *PKD2*, *TSC2*, and *BRSK2*). Terms are ordered based on significance (*p*-value). (**B**) Top 10 KEGG pathways of the expanded network based on the first neighbors of BRSK2 derived from the main PPI network. Terms are ordered based on significance (*p*-value).

Our attempts to understand the impact of the different mutations on the functions and interactions of proteins have revealed some interesting potential targets, one of which is the brain-specific serine/threonine-protein kinase *BRSK2* of the AMPK family. This protein is not heavily studied, and existing studies have linked *BRSK2* protein to neuron development; insulin secretion; and other biological processes such as apoptosis, cytoskeletal remodeling, and mTOR signaling [79,80,81]. The GO and KEGG pathway analysis of the first network, as revealed in Figure 4A, highlighted several significant findings that provided us with confidence in the notion that mTOR is a common pathway shared by *PKD1*, *PKD2*, *TSC2*, and *BRSK2*. Indeed, overactivation of the PI3K/Akt/mTOR pathway was found to encourage hyperproliferation in cancer and kidney cysts [40]. Furthermore, the GO and KEGG pathway results associated with the subnetwork focused on BRSK2 displayed the mTOR pathway as the most significant player, followed by some interesting findings such as autophagy, PI3K pathway, and cell cycle (Figure 4B). These findings suggest that mTOR and possibly other metabolic pathways, including PI3K and AMPK, as well as autophagy, may play an essential role in the disease pathogenesis and progression. Therefore, the newly recognized mutations in this work could tip the balance of these different cellular activities. The evidence used in STRING linking BRSK2 to TSC2 and PKD1 was based on text mining or co-expression; however, we conducted a text mining search using terminologies that are related to our work. Indeed, physical interactions between the examined proteins remain to be demonstrated in protein–protein interaction (PPI) studies [82]. Yet, following a similar strategy, we were able to obtain several encouraging data indicating BRSK2 is involved in the TOR pathway. Consequently, we hypothesized that the proteins TSC2, PKD1, and BRSK2 could be involved in the regulation of the mTOR pathway. Nevertheless, the extent to which this interaction is significant in the context of ADPKD pathogenesis or progression requires further experimental validation. Limitations of this study include the small sample size used and that protein biochemistry studies were not conducted. This study, along with other reports, is another authentication of ADPKD severity and incompatibility with life when both alleles of the *PKD* gene are affected [25]. The reported mutations were pathogenic and considered a causative mutation for the diseases.

## 5. Conclusions

Places that are known for high consanguineous marriage rates deliver unique populations for investigating genetically transmitted diseases, particularly autosomal recessive transmission. Herein, exome sequencing of DNA obtained from Saudi patients who presented with typical ADPKD revealed that mutations in *PKD1* and *PKD2* are the most common cause of typical ADPKD in this population. These patients displayed positive family history, early onset, and aggressive form of the disease. We also demonstrated other variants that presented with variable levels of disease severity. Patients with a positive family history of ADPKD might eventually receive substantial benefits from the advances in genetic screening tools to detect early-onset disease and overcome unpleasant consequences. Moreover, establishing early disease biomarkers that usually associate with an aggressive course can answer unresolved questions related to disease pathogenesis, prognosis, and therapeutic modalities. Genetic testing for ADPKD can improve diagnostic precision and prognosis as well as support family planning and genetic counseling. Further PPI and protein biochemistry studies to investigate the influence of these mutations on the *PKD1* and *PKD2* functions are required. In addition, large-scale population-based studies to verify these findings are recommended.

## Figures and Tables

**Table 1:** Demographic and clinical characteristics of the cases.

Patient ID	Age	Sex	Nationality	Ethnicity	Hypertension	eGFR (mL/min/1.73m^2^)	CKD stage	Affected Family Members	Other Diseases
P1	46	F	SA	Arab	Yes	NA	G4/A3	3	PKD
P2	37	M	SA	Arab	No	60–89 mL/min/1.73	G2/A1	2	Hereditary nephritis and CKD
P3	19	M	SA	Arab	No	15 mL/min/1.73	G5/A3	1	CKD
P4	18	M	SA	Arab	Yes	15	G5/A3	2	Hereditary nephritis CKD
P5	40	F	SA	Arab	Yes	60	G2/A3	1	CKD
P6	33	F	SA	Arab	Yes	5	G5/A3	1 and 6 carrier	CKD ESRD
P7	30	M	SA	Arab	Yes	60	G2/A2	1 and 6 carrier	CKD
P8	30	F	SA	Arab	Yes	60–89	G2/A3	2	CKD
P9	26	M	SA	Arab	No	49	G3a/A2	1	CKD
P10	26	M	SA	Arab	Yes	NA	G4/A3	0	CKD
P11	31	M	SA	Arab	No	NA	G5/A3	2	CKD ESRD

Patients are classified as G1-G5 based on the eGFR and A1-A3 based on the albumin:creatinine ratio (ACR). CKD, chronic kidney disease. PKD: Polycystic kidney disease. ESRD: End-Stage Renal Disease. GFR, glomerular filtration rate. SA, Saudi Arabia. eGFR: in males, 100–130 mL/min/1.73 m^2^; in females, 90–120 mL/min/1.73 m^2^.

**Table 2:** The mutations identified in all patients diagnosed with ADPKD. Table contains the mutated genes, mutations on the cDNA level and protein level, type of mutation, and exome. It also provides information about the zygosity and pathogenicity.

Patients	Gene	cDNA Change	Protein Change	Mutation Type	Exome No.	Zygosity	Pathogenicity	SNP ID
P1	*PKD1L1*	c.3813_3814delinsTG		Non-frameshift substitution	24	Hetero	Uncertain significance	
P2	*PKD1*	c.4264G > A	p.A1422T	Missense Variant	15	Hetero	Uncertain significance	rs140980374
P3	*PKD2*	c.1445T > G	p.F482C	Missense Variant	6	Homo	Pathogenic	rs75762896
P4	*PKD1*	c.1758A > C	p.E586D	Missense Variant	9	Hetero	Pathogenic	
P5	*PKD1L2*	c.404C > T	p.P135L	Missense Variant	2	Hetero	Uncertain significance	rs201455881
P6	*PKD2L2*	c.1364A > T	p.N455I	Missense Variant	9	Hetero	Uncertain significance	
P7	*TSC2*	c.5038C > T	p.R1680C	N/A	39	Exonic	Likely pathogenic	rs45517423
P8	*PKD1*	c.4264G > A	p.A1422T	Missense Variant	15	Homo	Uncertain significance	rs140980374
P9	*PKD1*	c.9774T > G	p.F3258L	Missense Variant	29	Hetero	Pathogenic	N/A
P10	None	N/A	N/A	N/A	N/A	N/A	N/A	N/A
P11	*PKD1*	c.4264G > A	p.A1422T	Missense Variant	15	Hetero	Uncertain significance	rs140980374

## Data Availability

All data generated or analyzed during this study are included in this published article.
